# A Molecular Analysis Provides Novel Insights into Androgen Receptor Signalling in Breast Cancer

**DOI:** 10.1371/journal.pone.0120622

**Published:** 2015-03-17

**Authors:** Jatin Mehta, Shailendra Asthana, Chandi Charan Mandal, Sunita Saxena

**Affiliations:** 1 National Institute of Pathology, ICMR, Safdarjang Hospital, New Delhi, India; 2 Department of Biochemistry, Central University of Rajasthan, Ajmer, India; University of Kentucky College of Medicine, UNITED STATES

## Abstract

**Background:**

Androgen Receptor (AR) is an essential transcription factor for the development of secondary sex characteristics, spermatogenesis and carcinogenesis. Recently AR has been implicated in the development and progression of breast and prostate cancers. Although some of the functions of the AR are known but the mechanistic details of these divergent processes are still not clear. Therefore understanding the regulatory mechanisms of the functioning of the AR in ER-/AR+ breast cancer will provide many novel targets for the purpose of therapeutic intervention.

**Methods/Results:**

Using bioinformatics tools, we have identified 75 AR targets having prominent roles in cell cycle, apoptosis and metabolism. Herein, we validated 10 genes as AR targets by studying the regulation of these genes in MDA-MB-453 cell line on stimulation by androgens like 5α-dihydrotestosterone (DHT), using RT-qPCR and ChIP assay. It was observed that all the identified genes involved in cell cycle except MAD1L1 were found to be up regulated whereas expression of apoptosis related genes was decreased in response to DHT treatment. We performed an exhaustive, rigid-body docking between individual ARE and DNA binding domain (DBD) of the AR protein and it was found that novel residues K567, K588, K591 and R592 are involved in the process of DNA binding. To verify these specific DNA-protein interactions electrostatic energy term calculations for each residue was determined using the linearized Poisson–Boltzmann equation. Our experimental data showed that treatment of breast cancer cells with DHT promotes cell proliferation and decreases apoptosis. It was observed that bicalutamide treatment was able to reverse the effect of DHT.

**Conclusion:**

Taken together, our results provide new insights into the mechanism by which AR promotes breast cancer progression. Moreover our work proposes to use bicalutamide along with taxanes as novel therapy for the treatment of TNBCs, which are positive for downstream AR signalling.

## Introduction

Androgen receptor (AR) belongs to a family of intracellular steroid hormone receptors that function as ligand dependent transcription factor which regulates target gene expression. The full length AR protein is a 110 kDa phosphoprotein, which mediates its physiological functions by binding to its ligand testosterone or after its conversion to 5α-dihydrotestosterone (DHT) by 5α-reductases. AR is responsible for mediating a myriad of physiological function of the androgens like male sexual development, spermatogenesis, maintaining bone mineral density, stimulating erythropoiesis, production of prostate-specific proteins and it also regulates several aspects of cell metabolism like lipid biosynthesis [1–3]. AR has an N-terminal transactivation domain, which contains the poly-Glutamine (CAG) repeat sequence, a DNA-binding domain (DBD) having two C4 type Zinc fingers, a hinge region and a C-terminal ligand-binding domain (LBD) which gets activated upon binding to androgens. Androgen binding to C-terminal of AR leads to the dissociation of chaperone proteins and dimerization of AR leading to a conformational change whereby its nuclear localization signal (NLS) is exposed. Exposed NLS then aids in the translocation of AR to the nucleus, where it binds to androgen-response elements (AREs) present in the promoters of several target genes in a tissue-specific manner. In the nucleus, AR recruits many other proteins, such as general transcription factors and RNA polymerase to the activate androgen-responsive genes [4]. Predominantly AR is known for activation of target genes, although recent evidences have emerged showing transcriptional repression by AR [5,6]. The transcription activity of AR is mainly regulated by bound coactivators and corepressors but AR might also regulate transcription by interacting with signal transduction proteins in the cytoplasm. A typical ARE consists of two hexameric half-sites arranged as inverted repeats with a spacer of 3bp separating the two half sites [7]. AR binds as a dimer to its cognate AREs in a sequence specific manner, upon ligand binding to regulate transcription. The consensus sequence for AR (GGT/AACAnnnTGTTCT) binding was deemed to be similar to the response element for glucocorticoid receptor (GR), progesterone receptor and mineralcorticoid receptor [8,9]. Mechanistic details of how AR uniquely binds and regulates the androgen responsive promoters is not yet completely understood. However, considerable variations in the AR binding sites have been observed in the ARE sequences of the target genes indicating that other regulatory mechanism might play a decisive role in AR mediated gene expression. Moreover, apart from the hormone response element (HRE), presence of enhancers, posttranslational modifications and protein-protein interactions are known to affect the target gene expression by the AR.

Breast cancer is a heterogeneous disease that encompasses a range of phenotypically distinct tumour types and accounts for 1.38 million new cases of breast cancer worldwide, with a mortality rate of more than 458,000 cases [10]. Traditionally, estrogen receptor (ER) and progesterone receptor (PR) are known to be the prominent players in the progression and development of breast cancer but recent evidences suggest an important role of AR in breast cancer progression as well [11,12]. AR plays a significant role in the development of normal mammary glands through the action of Wnt ligand [13]. Moreover, deranged Wnt pathway is involved in progression of breast carcinogenesis [14]. Recent evidences suggest that AR antagonizes ER function and plays an anti-proliferative role in ER+ve breast cancers whereas it plays a significant role in facilitating tumor cell growth in an androgen-dependent manner in an ER–/AR+ breast cancers [13,15]. Functional cross talks between the AR and HER2 signalling pathways in MDA-MB-453 and SUM-190 breast cancer cell have considerable similarities to prostate cancer cell lines suggesting an oncogenic potential of AR in ER–/AR+ breast cancers [11,16]. All these evidences suggest that understanding AR signalling will provide the rationale for targeting of different types of breast cancer cells for the purpose of therapeutic intervention.

In order to understand the genomic actions of the AR, the gene expression profiling of the putative AR targets was studied and the AR binding was shown using the ChIP-qPCR. Considerable amount of sequence variability was found in the individual AREs, prompting us to study the binding of the AREs and the AR DBD *in silico*, revealing the critical nucleic acid and protein residues required for this interaction to take place. Computational docking was performed in order to reveal key residues involved in the stabilization process of the nucleic acid and AR DBD. No assumptions of specific types of DNA-protein contacts were made, albeit, predictions of interactions were based on binding energies.

## Material and Methods

### Cell culture and reagents

Cancer cell lines MDA-MB-453 and LNCaP were obtained from cell repository at National Centre for Cell Sciences (NCCS), Pune, India. MDA-MB-453 cells were cultured in L15 media supplemented with 10% FBS (Gibco, USA)_,_ whereas LNCaP cells were maintained in RPMI 1640 medium. Androgen-regulation experiments were performed in trypsinized cells which were allowed to adhere to the culture flask overnight and then transferred into media supplemented with 10% charcoal-stripped FCS (BioAbChem, USA). The following day media was replaced with fresh charcoal-stripped complete medium supplemented with 10 nM of the DHT (Sigma-Aldrich, MO, USA) or vehicle control. Cells were harvested at various time point for the purpose of performing RNA isolation and ChIP experiments.

### Immunohistochemistry (IHC) staining

Immunohistochemistry (IHC) procedure were done as described earlier [17]. Briefly, freshly trypsinized cells were made into a homogenous suspension and a drop of cells was added onto a glass slide. Cells were air dried and fixed with cooled acetone. Paraffin embedded tissue sample was immersed in xylene for 15 minutes at room temperature (R.T.). Tissue sample and the cell were immersed in 90% ethanol, followed by 70% ethanol for 15 minutes each at room temperature. Endogenous peroxidase blocking was done using 3% H_2_O_2_ in methanol (PBS) for 30 minutes. Fixed cells and tissue were given 3 washes each with PBS and antigen retrieval was performed with 10mM citrate buffer in hot water bath for 10 minutes and 15 minutes respectively for the cells and the tissue samples. IHC was done on cells and tissue sections using 1:50 dilution of Her2 Antibody (Thermo Fisher Scientific, U.S.A.) and incubated overnight at 4°C. The tissue sections and the cells were washed with PBS, incubated with HRP conjugated secondary antibody for 1 hour at room temperature and developed with DAB (diaminobenzidine) as chromogen and hematoxylin staining was used as the counter stain.

### Identification of novel AREs by computational analysis

In the present study, we used an *in silico* approach to identify novel genes, which are directly under the transcriptional regulation of AR in breast cancer cell line MDA-MB-453. Genes having prominent role in cell cycle, apoptosis and metabolism were sourced from the cell cycle gene database (www.cyclebase.org, www.itb.cnr.it/cellcycle/‎), apoptosis gene database (www.deathbase.org) and metabolic gene database (www.humancyc.org) respectively. All the gene sequences were retrieved from the Ref_Seq database at National Center for Biotechnology Information [18]. Approximately 5kb upstream and downstream regions pertaining to the transcription start site (TSS) of the genes were taken from the respective data bases and used to search novel Androgen response elements (AREs) motifs having similarity to the consensus ARE sequence GGT/AACAnnnTGTTCT by using "TFSEARCH" (http://mbs.cbrc.jp/research/db/TFSEARCHJ). Scoring was based on the number of nucleotides in the query sequence that matched with the consensus sequence. The putative ARE sequence with the highest score was reported for each gene and cut off threshold score of 85 and the above were considered to be a significant hit.

### Reverse transcription (RT) quantitative PCR

Total RNA isolation, reverse transcription and RT-qPCR were done as described earlier [19–21]. MDA-MB-453 cells were seeded in 60mm culture plates and allowed to grow for 24 hrs, following which they were treated with 10nM DHT for 0, 4, 8, 12 and 24 hours. Vehicle treated cells were used as control. Cells were harvested, washed with PBS and lysed using 1ml of TRIzol (Invitrogen, Carlsbad, CA, USA). Cell pellets were gently pipetted several times and incubated for 5 minutes at room temperature (R.T.) to completely lyse cells. 200μl of chloroform (Sigma Aldrich, MO, USA) was added followed by vigorous shaking of the tubes and the samples were incubated for 3 minutes at R.T. Samples were centrifuged at 12000xg for 15 minutes at 4°C and the top layer was gently transferred into a new eppendorf. RNA was precipitated using 0.5ml of isoamyl alcohol and supernatant was carefully discarded. RNA pellet was washed with 1ml of 70% ethanol, dried and finally dissolved in 100μl of DEPC water. One microgram of the total RNA was used for the RT reaction in a final volume of 20 μl using the Superscript II First Strand RT synthesis kit (Invitrogen, Carlsbad, CA, USA) according to the manufacturer's instructions. 1ul cDNA was used to perform quantitative PCR (RT-qPCR) using SYBR Select Master Mix (Applied Biosystems, Foster City, CA) and gene specific primers (S1 Table) in ABI 7000 sequence detection system (Applied Biosystems, Foster City, CA). Standard curves were used to assess primer efficiency and average change in threshold cycle (ΔCT) values was determined for each of the samples relative to endogenous 18S rRNA (internal control) levels using the ΔΔCT method. Experiments were performed in triplicates to determine mean along with standard error, and student's t-tests were used to calculate statistical significant p values.

### Chromatin Immunoprecipitation (ChIP) Assays

Basic procedure of chromatin immunoprecipitation experiments was described earlier [22]. Chromatin immunoprecipitations were carried out using a kit following the manufacturer’s protocol (Millipore). ChIP experiments were performed in hormone-depleted cell lines in charcoal stripped FCS, following which the cells were treated with 10 nM DHT or vehicle for 24 hrs. 4x10^7^ cells were fixed with formaldehyde (Sigma Aldrich, MO, USA) at room temperature for 10 minutes. Reaction was stopped by adding glycine solution for 5 minutes at room temperature and two washes were given with ice-cold PBS (phosphate buffer saline). After the wash, cells were resuspended in PBS buffer having protease inhibitor cocktail II (provided along with the kit) and cells were collected by scraping them from dish into 15mL conical tubes and centrifuging at 720 x g at 4°C for 10 minutes. Supernatant was discarded and pellet was suspended in 80μl of ice-cold EZ-Zyme Lysis Buffer (supplemented with Protease Inhibitor Cocktail II). Then cells were lysed with lysis buffer by snap freezing in liquid nitrogen and thawing them on 37°C and the freeze-thaw cycle was repeated 3 more times. The cell nuclei thus extracted were subjected to chromatin digestion so as to give a smear of DNA prominently lying in range of 200–500bp by incubating at 37°C for 10 minutes with EZ-Zyme Enzymatic cocktail, provided with the EZ-Zyme Chromatin Prep Kit (Millipore). Positive control Anti-RNA Polymerase II antibody, negative control normal Mouse IgG (supplied along with the kit) or Androgen Receptor antibody ChIP Validated Antibody (Millipore) were added separately and incubated for 2 hour at 30°C followed by incubation with fresh Protein A agarose magnetic beads. Cleaved chromatin was immunoprecipitated using the Magna ChIP A/G kit (Millipore). Protein A/G magnetic beads were pelleted with the magnetic separator and supernatant was completely removed. Precipitated chromatin complexes were removed from the beads through 30-min incubation with 500 μl of elution buffer (1% SDS, 0.1 M NaHCO_3_). Finally, the protein–DNA cross-links were reversed by incubation at 65°C for 4 hours. ChIPed DNA fragments were purified using spin columns and the DNA fragments thus obtained were analyzed by qPCR DNA using 2μl of the ChIPed DNA, SYBR Select Master Mix (Applied Biosystems, Foster City, CA) and Chip primers (S2 Table). The fold enrichment was calculated by the normalization relative to the non specific Mouse IgG (supplied along with the kit).

### Analysis of ChIP DNA by real-time PCR

Input, IP and negative control normal Mouse IgG fractions were analysed by PCR with the respective primer pairs to amplify products of 150–250 bp in length, corresponding to either the promoter or internal coding regions (internal control) of the target genes. Internal control primers were taken at regions far from the site of the AR site in the coding region, where there no AR binding. Primer design was done by using primer 3 software, while using the default parameters were 23 nucleotides was the optimal primer length; primer *Tm* was between 60 and 70°C, with an optimal *Tm* of 68°C and maximal self-complementarity of 4.0 was allowed. Each of the reaction were run in triplicate and the dissociation curve was checked to verify that only a single species was amplified. Moreover, the value derived from the AR Quantitative ChIP data of the standard curve was expressed as "Fold change" where the sample value was divided by the average of 10 control values, multiplied by 100 and expressed as Fold change over IgG. List of primers used is mentioned in the S2 Table.

### Statistical analysis

Statistical differences were determined using the Student’s *t* test. P-value calculations were done using the Sigma Plot software and the resulting data are presented as the mean±SD (n ≥3). In all the experiments, P < 0.05 was considered to be statistically significant.

### Modelling Studies

#### Protein

The coordinates of the 3D structure of rat AR-DBD (PDB-ID 1R4I) were retrieved from RCSB Protein Data Bank (PDB) (www.rcsb.org/pdb/home/home.do). Refinement of the structure by energy minimization was done using molecular dynamics NAMD package with AMBER99SB force field [7,23,24]. The two steps of energy minimization: steepest descent and conjugant gradient were performed for 10,000 steps each. The simulation was conducted for 10 ns time slot in TIP3P solvent at a constant temperature of 300 K and a constant pressure of 1 atm and each component was coupled separately to an external bath using Beredson coupling method [25,26]. The lowest root mean square deviation (RMSD) structure of AR-DBD obtained from molecular dynamics (MD) was superimposed to starting structure (crystal 3D structure, PDB-ID 1R4I), and the RMSD value of 1.02 Å between them indicated, that these two structures are close to each other [data not shown].

#### DNA

Multiple Sequence Alignment was performed by ClustalW (http://www.ebi.ac.uk/Tools/msa/clustalw2/) and MUSCLE (https://www.ebi.ac.uk/Tools/msa/muscle/) programs by using their servers [27–29]. The structure of DNAs containing (the linear B-DNA fragments) with lengths of 15 bp were modelled by 3D-DART web interface [30]. While modelling DNA, the bend angle was chosen within a long range of 0^0^ to 60^0^ with tilt of 5^0^ as global changes.

The DOT docking tool was used to generate the complex of AR bound to its ARE. In the DOT calculation method, one molecule (the moving molecule) is systematically moved over the other molecule (the stationary molecule) in a complete translational and rotational search. Interaction energies for all configurations of the two molecules were evaluated as correlation functions, which were efficiently computed using the fast fourier transforms. The properties of both molecules are mapped onto grids. For each orientation of the moving molecule, the moving molecule is centred at each grid point and the interaction energy is calculated as the sum of the electrostatics and van der Waals terms. A cubic grid 128 Å on a side with 1-Å grid spacing (≈2.1 million points) and a set of 28,800 orientations for the moving molecule (≈7.5° spacing) gave over 60 billion configurations of the two molecules. The top several thousand configurations were retained. DOT also gives a complete grid of interaction energies with the energy mapped to the grid point at which the moving molecule is centred, so that the distribution of favourable energy configurations can be easily determined.

### Van der Waals energy term for the DOT calculation

The DOT van der Waals energy is proportional to the number of moving molecule atoms that lie within a favourable interaction layer surrounding the stationary molecule [31]. The shape potential of the stationary molecule is represented by an excluded volume, defined as all grid points inside the molecular surface calculated by the program msms, surrounded by a 3.0-Å favourable layer [32]. The shape of the moving molecule is represented by its atomic centres, including those of polar hydrogen atoms. Thus, a moving molecule atom can lie as close as a van der Waals radius from an atomic centre of the stationary molecule. This soft fit allows for grid effects and small conformational changes that may be induced upon intermolecular interactions. Each moving molecule atom that lies in the favourable region surrounding the stationary molecule contributes −0.1 kcal/mol to the van der Waals energy. A configuration is eliminated if any moving molecule atom lies within the stationary molecule’s excluded core.

### Electrostatic energy term for the DOT calculation

The electrostatic energy in DOT is calculated as the set of point charges representing the moving molecule placed within the electrostatic potential of the stationary molecule [33]. Partial atomic charges for the molecules were taken from the amber library that includes polar hydrogen atoms [34]. The stationary molecule was positioned on the cubic grid exactly as in the shape potential calculation. The electrostatic potential was then calculated with the program APBS, which uses finite-difference methods to solve the linearized Poisson–Boltzmann equation, thereby taking solvent and ionic strength effects into account [35]. A dielectric of 3 for the protein, a dielectric of 80 for the surrounding environment, an ion exclusion radius of 1.4 Å, and an ionic strength of 150 mM NaCl were used. The electrostatic potential of the stationary molecule was clamped so that the values at all grid points lie within the range of maximum negative and positive electrostatic potential values observed at the molecule’s solvent-accessible surface (out 1.4 Å from the molecular surface). This modification makes the electrostatic potential compatible with the approximate van der Waals potential. The ranges of the electrostatic potential were −1.5 to +3.5 kcal·mol^−1^·e^−1^ for AR-DBD as the stationary molecule. All the structures of DNA docked in the DBD binding site of AR-DBD, the analysis of its interactions, and the images were prepared by using VMD.

### MTT Assay

MDA-MB-453 cells (1X 10^3^/ well) were seeded in a 96 well plate (Corning, M.A., U.S.A) in L15 media with 10% FBS and they were allowed to adhere overnight. DMSO stock solutions of paclitaxel/5-fluorouracil and water solution of cyclophosphamide monohydrate were dissolved in culture medium to get the desired final concentrations. The following day media was replaced by media containing the indicated concentrations of the drugs, paclitaxel, 5-fluorouracil and cyclophosphamide (Sigma Aldrich, MO, USA) and the cells were grown for 48 hours at 37°C following the drug addition. In all the experiments involving DHT or bicalutamide (Sigma-Aldrich, MO, USA) treatment, tripsinized cells were allowed to adhere overnight in the 96 well plates. The following day, media was replaced with L-15 medium with 10% charcoal-stripped FCS (BioAbChem, USA) for 24 h followed by replacement of the media with fresh charcoal-stripped medium supplemented with 10 nM of the DHT (Sigma) or vehicle control for 24 hrs. After 48 hrs of incubation with drugs, MTT powder (Sigma-Aldrich, MO, USA) was dissolved 5mg/ml in L-15 without phenol red. The solution was filtered through a 0.2 μm filter and added to each culture well being assayed, to equal one-tenth the original culture volume and incubated for 3 hrs at 37°C. In all the experiments one set of wells, having only cells and medium, but no drug was taken as the control. Thereafter, the MTT containing medium was removed and the converted dye was solubilised with acidic isopropanol (0.1 N HCl in absolute isopropanol). Absorbance of converted dye was measured at a wavelength of 570 nm with background subtraction at 630nm using a multiwell spectrophotometer. The average values from triplicate samples was determined after subtraction of the average value for the blanks and the values thus obtained were plotted as a fraction of the control cells which were taken as 100% viable.

### Cell Growth Assay

The proliferation of MDA-MB-453 cells was evaluated by the MTT assay [36]. Log phase cells were trypsinized into single cell suspension and passaged into 96-well plates at a density 10^3^ per well and allowed to grow for different time durations in presence of DHT, bicalutamide or both. Vehicle treated cells were used as a control for the measurement of cell growth and proliferation. The growth rate was determined by MTT assay in triplicate, at the intervals of 12 hours each until the 5th day. For the assay, cells were grown and 20μl of 5mg/ml of MTT was added. Following the incubation the dye was solubilised as described earlier and the absorbance values so obtained were plotted after background subtractions with respect to the number of days of incubation. Calibration curves were obtained by plotting the reading of the averaged value per well with the time interval.

### Cell cycle analysis by flow cytometry

3 x 10^5^ cells were seeded in 35mm culture dish and allowed to adhere overnight. Cell cycle distribution and apoptotic cell death was determined by flow cytometric analysis. Cells were harvested after 48 hours of treatments with IC_50_ concentration of the various drugs as indicated, washed with PBS and resuspended in PI staining solution containing 3.8mM Sodium Citrate, 100 μg/ml propidium iodide, 0.3% Triton X-100 (v/v) and 20 μg/ml RNAse solution for 30 minutes on ice. Cell cycle distribution and DNA content was determined using the using FACS Aria II Special Order Research Product (Becton Dickinson, Franklin Lakes, NJ, USA) with an argon laser set to excite at 488 nm.

## Results

### Identification of novel ARE motifs

**Fig 1:**
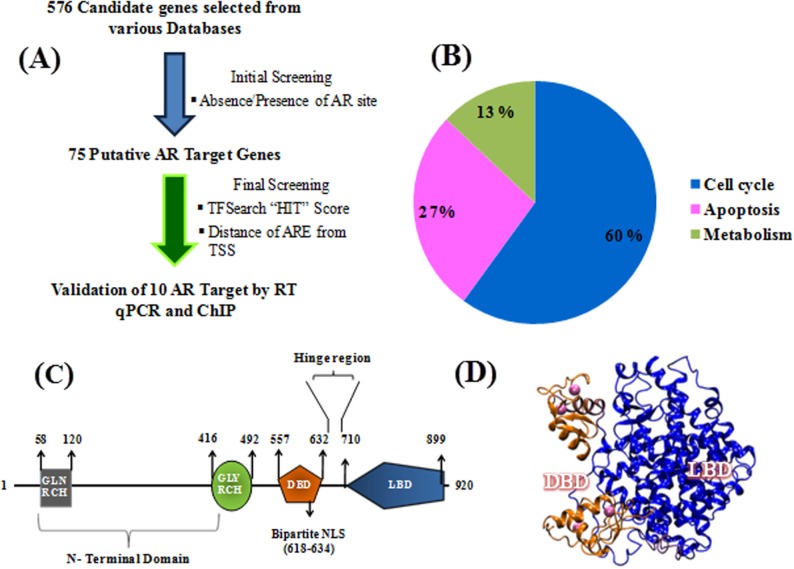
Strategy for the identification of novel androgen receptor regulated genes. (A) Flowchart of the methodology followed in finding novel AR targets. (B) Pie chart showing the distribution of the 75 putative AR targets in different cell processes. (C) Schematic diagram showing the various motifs and domains of the human androgen receptor; GLN RCH: Glutamine rich, GLY RCH: Glycine rich, DBD: DNA binding domain, NLS: nuclear localization signal, LBD: Ligand binding domain (D) The *in silico* model of AR containing both the LBD and DBD domain. The model is reproduced by applying the same protocol used by Helsen C. et al.[37] The crystal coordinates from the model of PPARy-RXRα heterodiamer (PDB ID 3DZY), the rat AR-DBD (PDB-ID 1R4I) was used to aligned onto the RXRα-DBD and the human AR-LBD (1XQ3) was aligned onto the PPARy-LBD using VMD tool. The human AR-LBD (2AM9), and rat AR-DBD (1R4I) was used for protein-protein docking using the program HADDOCK.

In order to gain better understanding of the androgen receptor (AR) regulatory networks, we sought to identify novel Androgen response elements (AREs) which are under the direct transcription control of androgen receptor. A total of 576 candidate genes having role in cell cycle, apoptosis and cellular metabolism were sourced from the cell cycle gene database (www.cyclebase.org, www.itb.cnr.it/cellcycle/‎), Apoptosis gene database (www.deathbase.org) and metabolic gene database (www.humancyc.org). Initial Prioritization of the genes selected for the purpose of analysis was based on the relation of the gene to AR function and role in carcinogenesis. In order to determine the presence of putative ARE(s), 5kb upstream and downstream of the transcriptional start site (TSS) of all these genes were scanned by using “TFSEARCH" version 1.3, transcription factor binding software. After initial screening of 576 genes, the resultant 75 hits having potential ARE(s) were tabulated. High scoring matches that were homo polymeric in the left or right half of the consensus sequence were excluded from the hits. Finally 10 genes were selected, on the basis of "TFSEARCH" hit score, gene expression and the distance of the putative AR binding sites from the TSS using a strategy shown in flowchart (Table 1, Fig. 1A). Majority of the AREs, 40 out of the total 75 genes were found in the upstream region whereas 31AREs were found downstream of the TSS (Transcription start site) and 4 genes had AREs present both upstream as well as downstream of their transcription start sites. It was observed that out of the total 75 putative AR targeted genes, approximately 60% had a role in cell cycle, 27% in apoptosis and 13% were found to be involved in the process of metabolism (Fig. 1B). Recent literature suggests that there is a cooperative binding between distant AR sites to regulate expression of a gene [9]. Moreover, individual AR binding at specific AREs is mediated by physical interactions between the DNA binding domain (DBD) and the ligand binding domain (LBD) of the nuclear receptor, which lie close to each other and are separated by only 78 amino acids (Fig. 1C). Inspite of this cooperative interaction between the DBD and the LBD, AR is known to recognise AREs which are quite deviating from the consensus sequence. We speculate that the physical interaction between the DBD and ARE might play a significant role in this process of ARE recognition by AR. In order to find all the important residues involved in the process of ARE binding, the coordinates of the 3D structure of rat AR-DBD (PDB-ID 1R4I) was taken and complete human AR DBD was recreated for the purpose of ARE docking (Fig. 1D) [37].

**Table 1:** Genes and the target ARE's regulated by androgen receptor.

Gene Name/ ID	Chromosome location	No of AREs/ TFSEARCH Score	ARE Sequence/ Position with respect to TSS (Transcription start site)	Gene function
AATK	17	1	5'GAGGAGTGGCGTGCA3’	Apoptosis: Increase Apoptosis and/or growth arrest [51,52]
(9625)		(90.1)	(4718 bp Downstream)	
ABL1	9	1	5'ACGCACCGCGGACTG3’	Cell Cycle: Increase cell division [37,39]
(25)		(88.1)	(1811 bp Upstream)	
BIK	22	1	5'TTACAGGCGCGTGCC3’	Apoptosis: Pro-apoptotic in function [49]
(638)		(86.4)	(3692 bp Downstream)	
BOK	2	1	5'GTGGGGCCGCGTGAC3’	Apoptosis: Induces apoptosis [48]
(666)		(85.5)	(4020 bp Downstream)	
CDT1	16	1	5'ACGCCATTCTCAGGC3’	Cell Cycle: Involved in formation of pre replication complex [40]
(81620)		(86.4)	(635 bp Upstream)	
ENDOG	9	2	5'TGCACGCGTACCACC3’	Apoptosis: Nuclease involved in DNA fragmentation during apoptosis [50]
			(546 bp Downstream)	
(2021)		(87.4, 87.3)	5’ACGCTATCGTCCGCG3’	
			(76 bp Downstream)	
KLF6	10	2	5'GTGGGGTTGCGTGCC3’	Cell Cycle: Its splice variants promote breast cancer metastasis and increased expression is found in tumors [41,42]
			(3448 bp Downstream)	
(1316)		(97.5, 86.9)	5'TTGGCATTGCGTGAA3’	
			(2172 bp Downstream)	
LIPH	3	1	5'TTCACGCGATTCTCC3’	Metabolism: It is a member of mammalian triglyceride lipase family [47]
(200879)		(87.4)	(1502 bp Downstream)	
MAD1L1	7	1	5'CACGCCACCACATCT3’	Cell Cycle: It plays a role in cell cycle control and tumor suppression [45,46]
(8379)		(87.7)	(1460 bp Downstream)	
SGOL2	2	1	5'TAGGGACTGCGTGCC3’	Cell Cycle: It promotes chromosomal biorientation and chromosomal segregation in cell division [43,44]
(151246)		(89.4)	(476 bp Downstream)	

### Validation of gene expression and AR binding upon androgen stimulation

**Fig 2:**
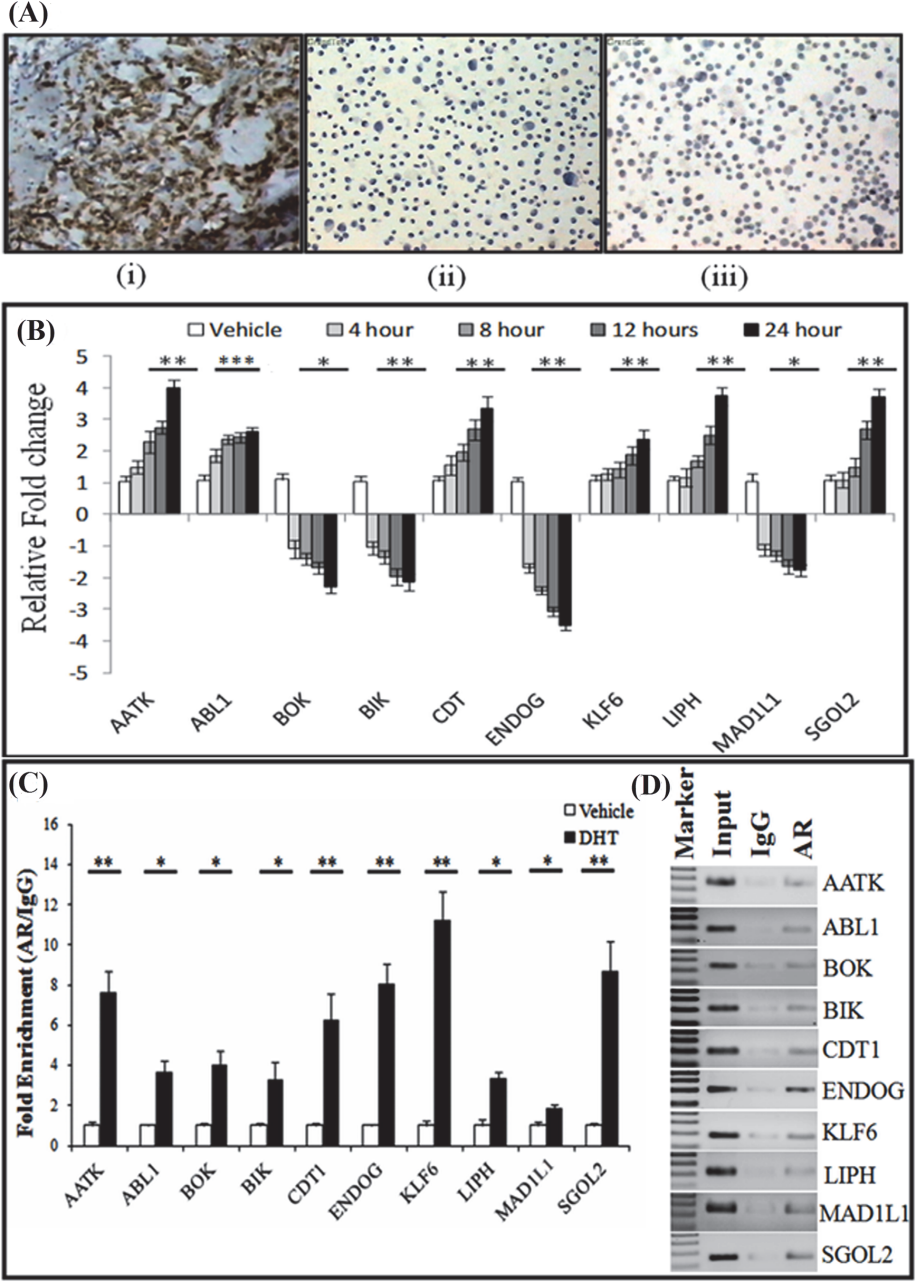
Androgen regulation of previously uncharacterized genes in Her2 negative MDA-MB-453 cells. (A) Surface Her2 expression was checked using the IHC staining of (i) A Her2 positive axillary lymph node having breast cancer metastasis was taken as the positive control (ii) MDA-MB-231 cells were taken as negative control (iii) MDA-MB-453 cells showing negative staining for Her2. (B) MDA-MB-453 cell were steroid starved for 24 hrs and then they were treated with 10nM/L of DHT or vehicle control for the indicated time points. Total RNA was isolated and relative mRNA levels were analyzed by RT-qPCR for the indicated genes. (C) ChIP assay were performed with AR antibody or control IgG antibody in MDA-MB-453 cells treated with 10nM/L of DHT or vehicle control for 24 hrs. The fold enrichment of coprecipitating DNA was determined by qPCR for the indicated promoters. Error bars are means ± SD of three independent experiments; (*p < 0.05, **p < 0.01, ***p < 0.001). (D) Standard PCR for the AR, IgG and input DNA was performed for the indicated genes.

Gene expression profiling of the AR targets was done in MDA-MB-453 breast cancer cell line after steroid depletion for 24 hrs followed by stimulation with DHT for 4, 8, 12 and 24 hrs. *MDA-*MB-453 cancer cell line was chosen as the breast cancer model as it is a triple negative cell line having ER–/AR+ phenotype and it was experimentally verified that Her2 expression is not there in MDA-MB-453 cells by IHC staining (Fig. 2A). It was observed that upon DHT treatment, the genes involved in cell cycle were up regulated, whereas all the genes except AATK, involved in the process of apoptosis were down regulated (Fig. 2B).It was further observed that all the genes showed maximum regulation by AR at 24 hours of time point. We identified that cell cycle related genes like ABL1 (p = 0.0001), CDT1 (p = 0.006), KLF6 (p = 0.005) and SGOL2 (p = 0.0011) had up-regulation greater than 2 fold upon DHT stimulation as compared to the vehicle treated cells (Fig. 2B). ABL1 is a non receptor protein tyrosine kinase that functions as a protooncogene and plays important roles in the processes of cell differentiation, cell division and cell adhesion [38,39]. Moreover, Abl tyrosine kinase upon activation is known to regulate expression of genes to promote S phase progression [40]. CDT1, a gene involved in the formation of pre replication complex along with KLF6 were found to be significantly up regulated in DHT treated MDA-MB-453 cells [41]. Our RT-qPCR data showed that treatment of MDA-MB-453 cells with DHT increased KLF 6 mRNA expression in comparison to untreated cells (Fig. 2B). KLF6 and its transcript variants have been implicated in the breast cancer metastasis and it is found to be up regulated in many tumors [42,43]. SGOL2 is a gene involved in chromosomal disorientation and chromosome segregation during cell division [44]. Furthermore, knockdown of SGOL2 via small interfering RNA has been shown to induce kinetochore attachment defects and delayed entry into anaphase [45]. Herein, we show that DHT treatment increased SGOL2 gene expression, suggesting a role of AR in cell cycle progression (Fig. 2B). Surprisingly, MAD1L1(p = 0.04) was the only cell cycle related gene involved in the process of checkpoint progression that was 1.7 fold repressed upon DHT treatment. Mutations in MAD1L1 have been found in cancers of prostate, breast, brain and lungs and over expression of wt-MAD1L1 is known to inhibit cell growth, thus suggesting a tumor suppressor function of MAD1L1 [46,47]. Our data indicates that AR may increase cell cycle progression by down regulating MAD1L1 gene expression. LIPH was the only member studied involved in the process of lipid metabolism and it was found to be 3.7 fold (p = 0.0023) up regulated on DHT stimulation. LIPH belongs to mammalian triglyceride lipase family and catalyses the formation of 2-acyl lysophosphatidic acid (LPA), which is a lipid mediator, involved in diverse biologic processes, like platelet aggregation, smooth muscle contraction, and stimulation of cell proliferation [48]. We found out that BOK (p = 0.03), BIK (p = 0.004) and ENDOG (p = 0.0012) were some of the genes involved in the process of apoptosis which were repressed more than 2 fold on DHT treatment [49–51]. AATK (apoptosis-associated tyrosine kinase) was the only apoptosis inducing gene found to be 4 fold (p = 0.0018) up regulated in MDA-MB-453 cell on 24 hrs of DHT stimulation as shown in Fig. 2B [52,53]. This apparently contradictory result noted that AATK may have different role in breast cancer cell line MDA-MB-453.

In order to validate the AR binding to the individual AREs, we performed real-time PCR (qPCR) using ChIPed DNA fragments as template to prove the physical occupancy of AR to these promoters upon DHT stimulation (Fig. 2B). We were able to confirm AR binding to AREs of multiple promoters, including AATK (p = 0.006), CDT1 (p = 0.014), ENDOG (p = 0.005), KLF6 (p = 0.004) and SGOL2 (p = 0.009) having more than 6 fold promoter occupancy as compared to untreated cells (Fig. 2C-D). Interestingly, except for ENDOG, AATK, SGOL2 and CDT1 promoter occupancy did not coincide with concomitant increase/decrease in expression as reflected by RT-qPCR suggesting that presence of enhancers, epigenetic mechanism or post transcriptional regulation such as mRNA stability or involvement of microRNAs may play an important role in the expression of these individual genes in response to DHT stimulation. Moreover, the results of AR binding were further validated in LNCaP cell and similarity of the results indicated that these genes are regulated by AR universally and not in a tissue specific manner (S1 Fig.).

### AR binding to the AREs

**Fig 3:**
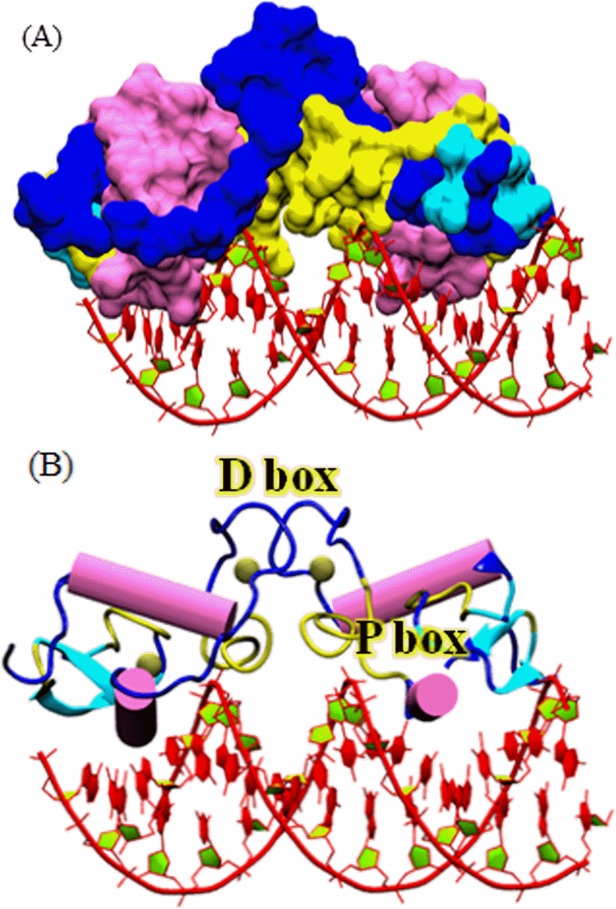
DBD domain organization of androgen receptor. (A) Surface representation of the rat AR-DBD structure bound to the ARE (Androgen response element). (B) Cartoon representation of AR-DBD bound to ARE. The α helix in pink, beta sheets in cyan, loops and turns are in blue and yellow color, respectively. Zinc ions are presented as grey spheres.

The DNA binding domain (DBD) is a highly conserved domain among the nuclear receptors (NRs). The AR-DBD contains two conserved motifs namely, P box (GSCKV), which interacts with the major groove of the DNA, while the second motif, D box (ASRND), which play a role in DBD-mediated AR dimerization. Surface representation of AR binding to its ARE is illustrated in Fig. 3A. DBD domain of AR contains two zinc finger motifs, where each metal ion is coordinated by four cysteine residues (Fig. 3B). There is only one crystal structure available for the rat AR-DBD, which was resolved in a complex with a DR3 response element (PDB code: 1R4I) [7]. According to the reported crystal structure, the AR-DBD is formed by two short anti-parallel beta-strands and two perpendicular alpha-helices [54]. This organization allows the AR-DBD to bind to the DNA in the form of a “head to head” dimer, where one monomer binds the half-site response element with high affinity and the second binds the other half-site with lower affinity [7,54].

### Docking of DNA on AR-DBD

**Fig 4:**
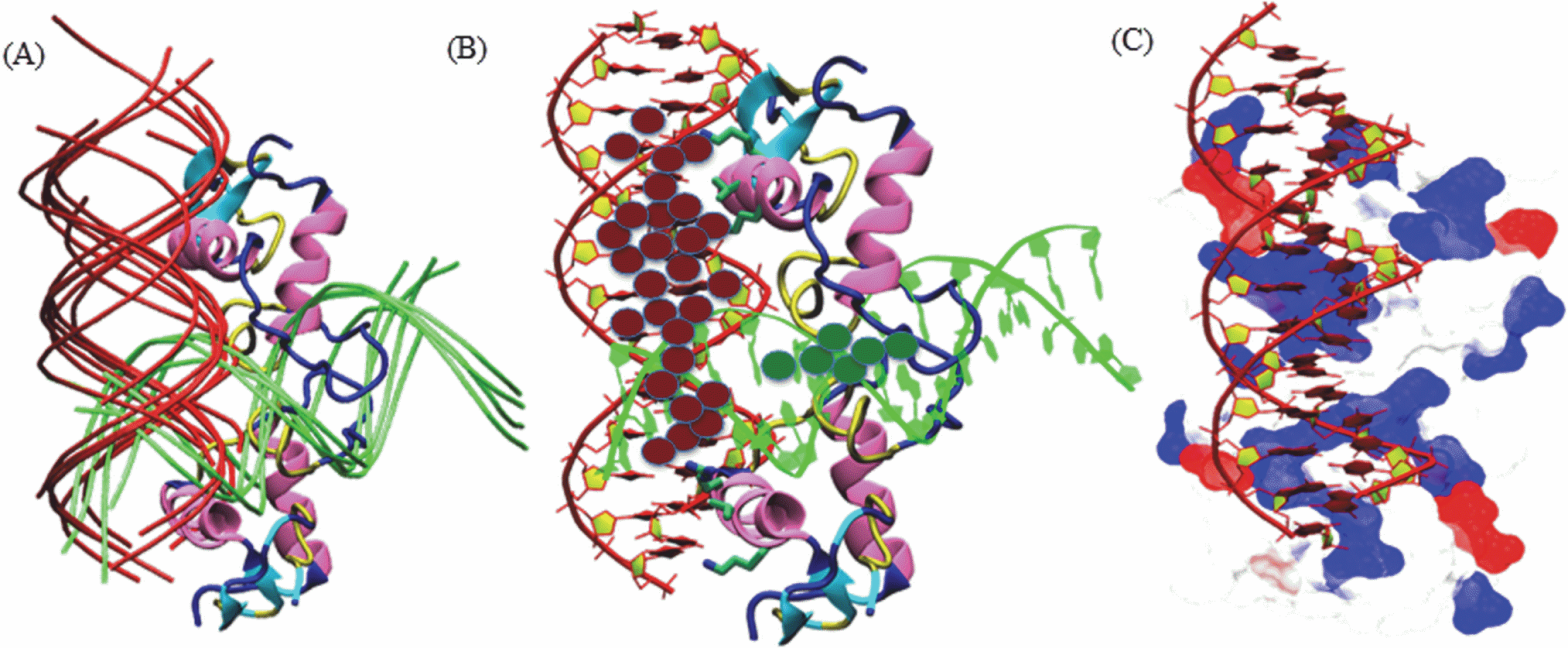
Docking of Linear B-DNA fragments to AR-DBD. (A) AR-DBD showed two distinct DNA- binding sites. The DNA fragments docked at site I in the top ranked solutions (shown by their phosphate backbone, red), and the DNA fragments docked at site II (in green color) shows similar orientations. (B) Top 200 ranked solutions (first site in red and, second site in dark green spheres) found by DOT clusters at site I (representative docked DNA fragment, rank I, red DNA backbone), and site II (rank 3, green backbone). (C) Electrostatic surface view of the best conformer of DNA docked at AR binding site.

In order to understand how the extensive positive potential created by androgen receptor influences DNA binding, we performed rigid-body dockings with DOT, a computational docking tool for macromolecular interactions [31]. Studies on winged-helix transcription factors, nucleosomes and linker shows that DOT is an effective tool for predicting protein–DNA interactions [55]. Docking of linear B-DNA (15 bps) (the moving molecule) to AR (the stationary molecule) identified two distinct DNA-binding sites in the 200 top-ranked solutions (Fig. 4A and 4B), with each site represented in the top 50 solutions. Our docking using B DNA separates the primary site into sites I and II (red and green in Fig. 4B). The B-DNA solutions, which are docked at site I (31 of the 50 solutions) showed significant interactions with the protein residues of AR protein. It was observed at site I that side chains of highly conserved amino acids of AR (Lys-563, Val-564 and Arg-568) were found inserted into the DNA major groove, while the backbone of some other residues of AR (Lys-567, Lys-588, Arg-591 and Lys-592) were also found having high affinity contact with B-DNA. Other B-DNA solutions, which docked at site II (13 among top 50 solutions), were not found to be having significant affinity with AR (Fig. 4A). The localization of site II between the P-boxes (far away from key residues) and due to the low binding affinity (data not shown), we concluded that site I is the prime site, and further analysis was conducted on site I only Additionally, we also generated and mapped the electrostatic potential of AR-DBD interaction with B-DNA as electrostatic interactions mediate bulk of DNA-AR binding (Fig. 4C). It was observed that the complementary interactions between AR and DNA fit well along with the basic patches of AR.

### Sequence Alignment of the AREs

**Fig 5:**
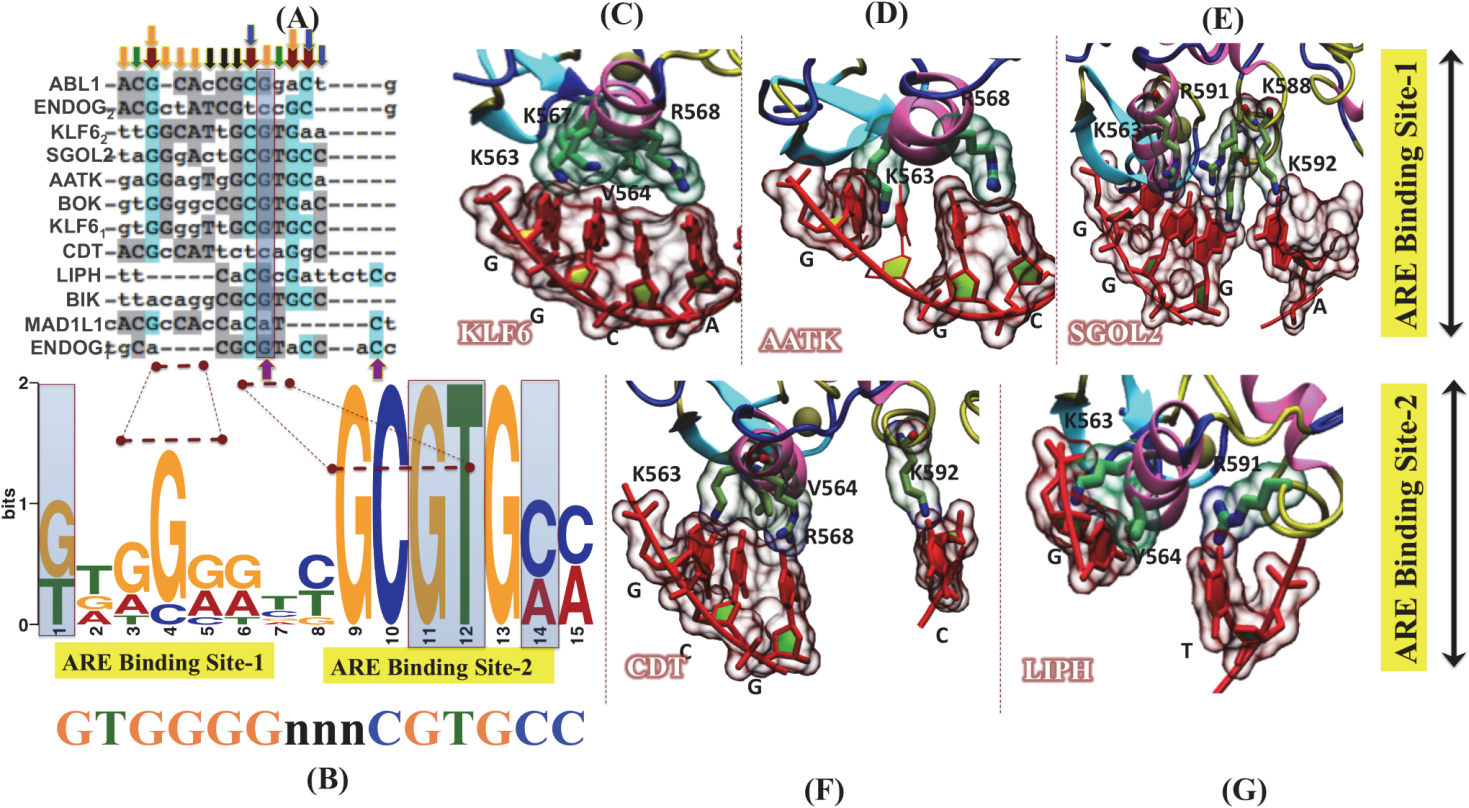
Interaction map showing the complexes formed by docking of ARE(s) to AR DBD. (A) Multiple sequence alignment of selected ARE genes. Novel conserved residues identified from literature are marked by deep red arrows, whereas the two purple arrows (at the bottom of the alignment) indicate the highly conserved contact points between ARE-DBD and cognate AREs [9]. All ARE sequences shown were analyzed using Clustal W followed by glam2scan tools. All the small arrows mentioned at the top of panel A are showing the resemblance of the outcomes between sequence alignment and glam2scan. (B) The graphical representation of the consensus sequence generated through MEME software, where the height of the letters indicates the frequency of each base. In the consensus ARE sequence, dotted lines are used to highlight those nucleotide residues which actually involved in interactions is showing the ARE interaction site-1. (C-E) Docking results showing the ARE interaction site-1. (F-G) Docking results showing the ARE interaction site-2. The molecular basis for the interaction of B-DNA strands of AREs and the key residues of AR-DBD. The interacting protein residues are shown in green liquorice with transparent surface, while nucleotides are rendered in red liquorice with transparent surface.

Sequence alignment of the AREs present in all the 10 genes (Table 1) under study was performed in order to find the conserved set of nucleotides involved in the DNA-protein interactions. The sequence alignment clearly revealed that there were six set of nucleotides, which were found to be conserved in all the AREs, which are marked by brown and green arrows (Fig. 5A). Sequence alignment of the AREs reveals that the guanine at position 3 is the conserved residue for most sequences (Fig. 5A). It was observed that conserved residues in position 11 and 14 were found in agreement with the work of Kimberley R. et al. (indicated by green arrows in Fig. 5A), while some new set of conserved residues were observed at positions 3, 10, 13 and 14 (Marked by brown arrows). Moreover, a PWM showing the results of the alignment, showed a consensus sequence having the sequence ""GTGGGGnnnCGTGCC" was also observed (Fig. 5B). Overall, the sequence alignment clearly showed that the majority of the conserved residues were either nucleotide G or nucleotide C.

### Interaction of AR-DBD with the ARE(s)

In order to decipher the DNA-protein contact points between the AR-DBD and ARE(s), 5 genes namely, AATK, CDT1, KLF6, LIPH and SGOL2 were selected on the basis of TF search score, fold enrichment in ChIP-qPCR experiments for the purpose of interaction studies. As mentioned earlier, the molecular interaction maps of DNA-AR supports well the outcome of alignment results, as it was showing interactions with the key residues of P-box namely K563, V564, R568 [7]. Our data shows that novel residues K567, K588, R591 and K592 make important contributions in increasing the stability of DNA-protein complexes (Table 2, Fig. 5). It was observed that in KLF6 ARE, the interacting residues K563, V564, K567 and R568 were binding with GGCA set of B-DNA (Fig. 5C). Overall, the total interaction energy between the AR and the SGOL2 ARE was-13.4 kcal/mol, while the overall binding energy between AR and AATK was-8.1 kcal/mol. It was found that the interaction of two amino acid (G@K563, G@R568 and C@R568) with three nucleotides: GGC were mainly involved in stabilizing the interactions between AR-DBD and AATK ARE (Fig. 5D). The overall interaction energy between AR and KLF6 was found to be-19.2 kcal/mol (Table 2). The residue wise decomposition explored the residue wise contributions. It was observed that residue A@R568 contributed maximum with the energy-5.9 kcal/mol whereas other residues C@K567, G@V564, and G@K563 contributed-3.2, -1.8, and-2.2 kcal/mol, respectively. In case of SGOL2, the major interaction was established between A@K592 with energy of-4.8 kcal/mol. The other key residues involved in AR-DBD and SGOL2 ARE interaction were G@K563, G@K588 and G@R591 with an energy-2.7, -2.2 and-1.7 kcal/mol respectively (Table 2, Fig. 5E). The residue wise energies were-2.8, -2.6 and-1.2 kcal/mol. The total binding energy between AR and LIPH was-8.9 kcal/mol (Table 2, Fig. 5G). The major contribution to establish the stability between AR and LIPH was mainly contributed by three amino acids G@K563, G@V564 and T@R591 with energies-2.3, -1.6 and-4.7 respectively (Table 2, Fig. 5G). In case of CDT1 the overall binding energy was found to be-9.2 kcal/mol (Fig. 5F). It was observed that G@K563, C@V564, C@R568 and C@592 were main residues with energy-0.7, -1.1, -3.8 and—2.3 kcal/mol respectively. This result clearly showed that the binding affinity of KLF6 and SGOL2 were higher than other AREs, while AATK, CDT1 and LIPH showed similar kind of interaction pattern. This outcome of docking was evident from the result of alignment as KLF6 shared 80% similarity with SGOL2. Secondary, the common key residues of B-DNA (mostly G and C) were among those residues, which are conserved in alignment, which reveal a nice agreement between the outcomes.

**Table 2:** Details of the gene interaction map.

Genes	Residues	Residue wise interaction energy (Kcal/mol)	Nucleotide residue	Total binding free energy
KLF6	K563	−2.2 ± 0.7	GGCA	−19.2
	V564	−1.8 ± 1.1		
	**K567**	−3.2 ± 1.2		
	R568	−5.9 ± 2.1		
SGOL2	K563	−2.7 ± 1.8	GGAG	−13.4
	**K588**	−2.2 ± 0.8		
	**K592**	−4.8 ± 1.7		
	**R591**	−1.7 ± 1.3		
AATK	K563	−2.8 ± 1.1	GGC	−8.1
	R568	−2.6 ± 2.1		
LIPH	K563	−2.3 ± 2.2	GT	−8.9
	V564	−1.6 ± 0.9		
	**R591**	−4.7 ± 3.1		
CDT	K563	−0.7 ± 1.2	GCCC	−9.2
	V564	−1.1 ± 1.7		
	R568	−3.8 ± 2.1		
	**K592**	−2.3 ± 2.4		

The key residues involved in interaction of AR DBD with ARE were K563, V564, **K567**, R568, **K588, R591, K592**. The newly identified residues are highlighted in bold.

### AR down regulates apoptosis and promotes cell proliferation on DHT stimulation

**Fig 6:**
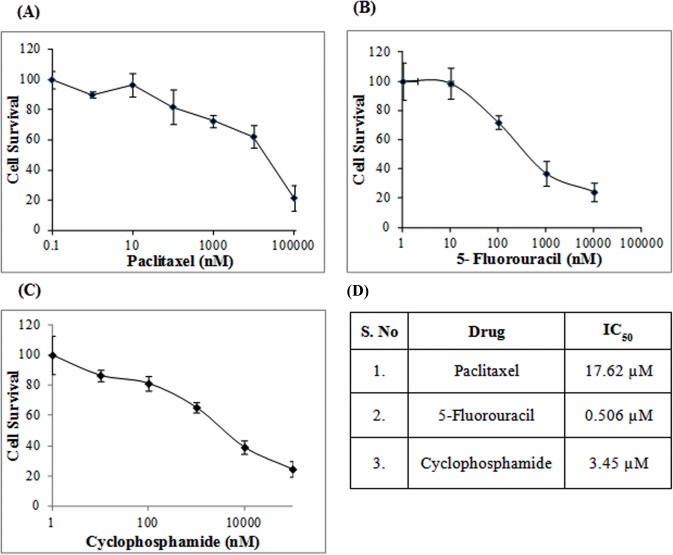
Drug resistance of MDA-MB-453. (A-C) MDA-MB-453 cells were treated with the indicated concentration of anti-cancer drugs paclitaxel, 5-fluorouracil and cyclophosphamide respectively and the cell survival was determined using MTT assay. (D) Table showing the IC_50_ concentration of drugs determined using the survival curves. Error bars are means ± SD of three independent experiments; (*p < 0.05, **p < 0.01, ***p < 0.001).

**Fig 7:**
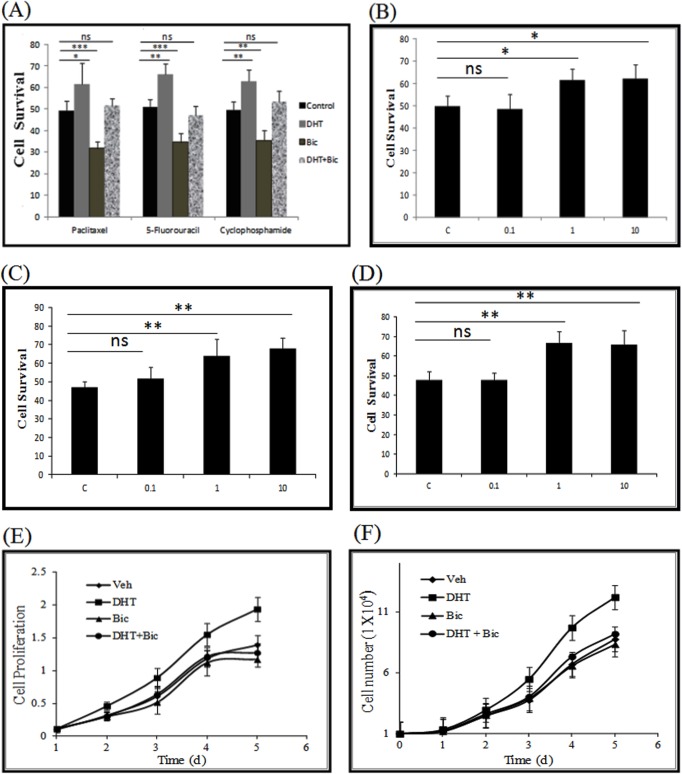
DHT stimulation decreases apoptosis and induces cell proliferation in MDA-MB-453 cells. (A) Effect of DHT stimulation on cell survival caused by paclitaxel, 5-fluorouracil and cyclophosphamide in presence of 10nM/L of DHT (+) or vehicle control (-) for 24 hrs. Error bars are means ± SD; (ns: not significant,*p < 0.05, **p < 0.01, ***p < 0.001). (B-D) Increased cell survival cause by concentration dependent increase in DHT (nM/L) in presence of paclitaxel, 5-fluorouracil and cyclophosphamide respectively. (E) Role of AR in regulating MDA-MB-453 cell proliferation on DHT stimulation. Monolayer growth rates of cells were determined by the MTT assay and 1.39 fold difference (P value = 0.0154) was observed between vehicle and DHT treated cells (F) Live cell counting was done with trypan blue done to estimate cell the survival. DHT treated cells showed 1.55 fold (P = 0.003) difference over vehicle treated cells. All the mean and standard deviation are obtained from three independent experiments. Error bars are means ± SD of three independent experiments; (*p < 0.05, **p < 0.01, ***p < 0.001).

In order to observe the effects of DHT on cell apoptosis, we selected three well known drugs namely paclitaxel, cyclophosphamide and 5-fluorouracil used in chemotherapy of breast cancer. Cyclophosphamide and 5-fluorouracil along with methotrexate are commonly used as CMF (cyclophosphamide, methotrexate and 5-fluorouracil) adjuvant and neoadjuvant chemotherapy for the treatment of breast cancer [56,57]. Paclitaxel (taxol) is a member of the taxane class of agents, that targets microtubules and induces apoptosis and it is currently used for the treatment of a wide range of carcinomas including breast, ovarian and non-small cell lung cancers [58]. In order to access the fate of the MDA-MB-453 on DHT treatment followed by addition of chemotherapeutic drugs, cell survival was measured using MTT assay. To study the effect of AR on apoptosis, IC_50_ values of the chemotherapeutic drugs paclitaxel, 5-fluorouracil and cyclophosphamide were determined to be 17.62μM, 0.506 μM and 3.45 μM respectively (Fig. 6. A-D). The effect of AR on the process of apoptosis was studied by adding IC_50_ concentration of the paclitaxel, 5-fluorouracil and cyclophosphamide drugs in presence of DHT, bicalutamide (AR antagonist) or both. It was observed that there was a significant increase in the cell survival on DHT treatment as compared to the vehicle treated cells, even in presence of IC_50_ concentration of the 3 therapeutic drugs, suggesting that AR reverses the chemotherapeutic drug induced apoptosis in MDA-MB-453 cells (Fig. 7A). We observed that paclitaxel (p = 0.01) induced apoptosis was reversed by DHT treatment (Fig. 7A). Similar decrease in apoptosis was observed in the presence of 5-fluorouracil (p = 0.002) and cyclophosphamide (p = 0.006) on DHT stimulation (Fig. 7A). We speculate that AR might be involved in the process of resistance to these chemotherapeutic agents. Moreover, the cell survival was significantly decreased in the presence of bicalutamide alone as compared to the vehicle treated cells, with paclitaxel (p = 0.0005) having a highly significant decrease in cell survival caused by increase in chemotherapeutic drug induced apoptosis followed by 5-fluorouracil (p = 0.0009) and cyclophosphamide (p = 0.003) (Fig. 7A). It can be concluded that DHT addition makes the cell resistant to apoptosis and this is reversed by the addition of bicalutamide, which acts synergistically with the chemotherapeutic agents to induce cell death. Interestingly, addition of both DHT and bicalutamide, in the presence of paclitaxel, 5-fluorouracil and cyclophosphamide seemed to cancel each other's effect having an insignificant difference in cell death as compared to the vehicle treated cells (Fig. 7A). In order to be sure that the increased cell survival owing to decreased chemotherapeutic drug induced apoptosis was due to the direct actions of AR, we observed the effect of increasing concentration of DHT on cell survival in presence of IC_50_ concentrations of the drugs paclitaxel, 5-fluorouracil and cyclophosphamide respectively (Fig. 7B-D). An increase in cell survival was seen in a concentration dependent manner on stimulation with DHT in presence of paclitaxel, 5-fluorouracil and cyclophosphamide addition respectively suggesting that DHT stimulation decreases cell death and promotes cell survival (Fig. 7B-D). Further, the proliferation of MDA-MB-453 cells on DHT treatment was observed both by the methyl thiazolyl tetrazolium (MTT) assay and live cell counting (Fig. 7E-F). MDA-MB-453 cells showed increased growth responsive upon DHT stimulation and this increased cell proliferation was abolished by treatment with non steroidal AR antagonist, bicalutamide (Fig. 7E). There was 1.39 fold (p = 0.0154) increase observed on DHT stimulation on the cell growth as compared to the vehicle treated cells, which was abrogated upon addition of bicalutamide. It was observed that bicalutamide treated cells showed a statistically insignificant change in cell growth in comparison to the vehicle treated cells. Similar increase in viable cell number was observed on cell counting by trypan blue. DHT treated cells showed a 1.55 fold (p = 0.003) increase over vehicle treated cells. Taken together our data suggest that there is positive correlation between AR function and cell proliferation in presence of DHT. Moreover, AR stimulation by DHT leads to decrease in cell death in presence of chemotherapeutic agents, indicating a chemo preventive role of AR.

**Fig 8:**
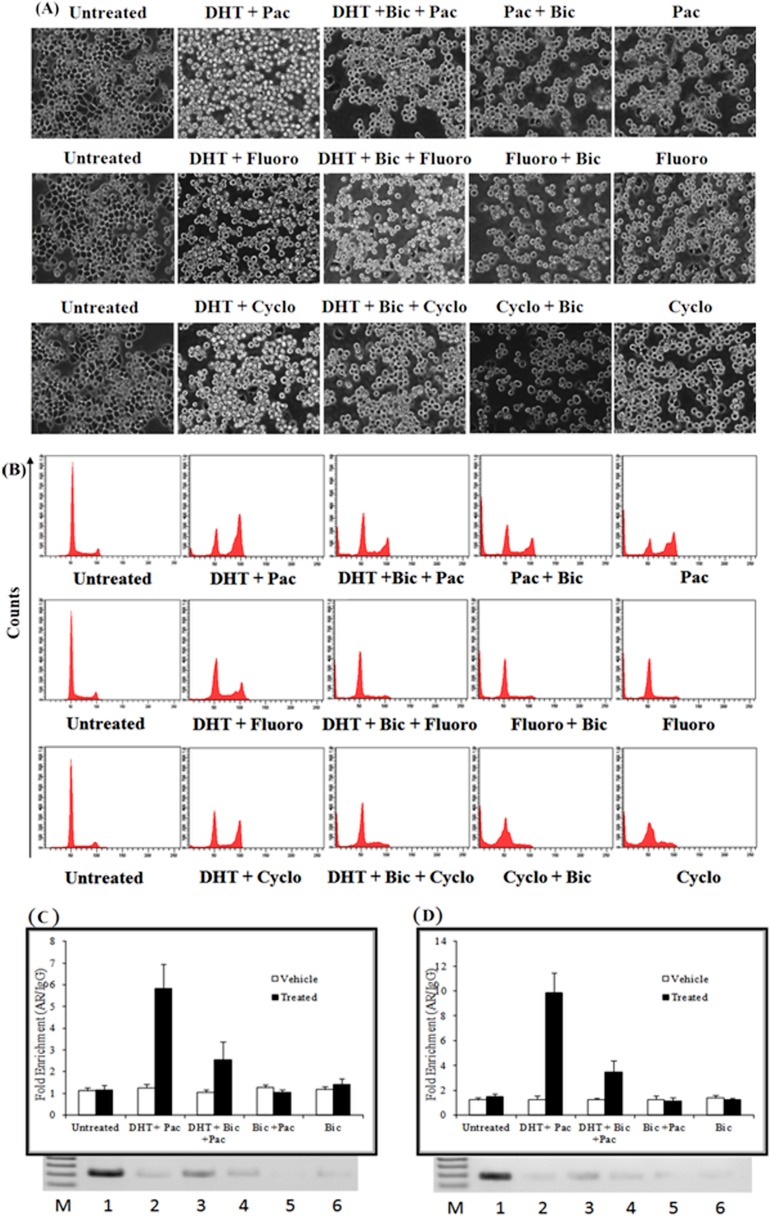
DHT mediates cell proliferation and Bicalutamide (Bic) abrogates this effect. (A) MDA-MB-453 cells treated with indicated treatments for 48 hours and their morphology was observed at higher magnification and randomly selected microscopic fields were photographed. (B) 3 x 10^5^ cells were plated in 35mm dish and the cell were treated with IC_50_ concentrations of the various drugs and harvested at 48 hours for DNA content analysis by flow cytometry. **(C)** AR binding to the promoter of CDT and KLF by ChIP-qPCR showing the binding of AR to CDT promoter in presence of Paclitaxel and various other treatments.(D) ChIP-qPCR showing the binding of AR to KLF promoter in presence of Paclitaxel and various other treatments. The gel panel below the picture shows the standard PCR validation of the result. All the means and standard deviation are obtained from three independent experiments. Pac: Paclitaxel; Bic: Bicalutamide; Cyclo: Cyclophosphamide; Fluoro: 5'-Fluorouracil, M: Marker, 1: Input, 2: Untreated control, 3: DHT +Pac,: 4: DHT+ Bic +Pac, 5: Bic + Pac, 6: Bic.

It was observed that DHT induces AR mediated cell proliferation even in the presence of IC_50_ concentration of the drugs, suggesting a chemopreventive effect of AR (Fig. 8 A and B). Bicalutamide addition along with DHT reversed the cell proliferation by decreasing the population of G2-M cells as compared to DHT alone (Fig. 8B). Moreover, bicalutamide in presence of various drugs, induced apoptosis and cell death was more, as compared to the drug alone (Fig. 8A and B). It has been reported that paclitaxel induces G2-M growth arrest followed by the accumulation of sub-G1 (apoptotic) cells [59]. We observed that treatment of MDA-MB-453 cells resulted in both G2-M arrest and cell proliferation in the presence of paclitaxel and DHT treated cells (Fig. 8B). It was observed that the sub-G1 (apoptotic) population was considerably less as compared to cells treated with paclitaxel alone. Moreover, the DHT mediated cell proliferation was also observed in the presence of the drugs cyclophosphamide and 5'-Fluorouracil. It has been reported that AR plays an important role in therapeutic resistance in prostate cancer [60].

It was observed that DHT mediated AR binding to the promoters of CDT and KLF was abrogated on the addition of bicalutamide (Fig. 8C-D and S2 Fig.). Moreover, the binding AR was completely stopped on addition of bicalutamide or the IC_50_ concentration of the drug. Bicalutamide is known to prevent nuclear localization of AR and therefore no binding was observed in the presence of bicalutamide in combination with a drug or bicalutamide alone (Fig. 8C-D and S2 Fig.). However, we observed binding in presence of the drug, DHT and bicalutamide suggesting that binding was due to some residual AR present in the nucleus. Taken together these data suggest that AR binds and directly up regulates the cell cycle genes like CDT and KLF and causes the cell proliferation in MDA-MB-453 cells (Fig. 8). Moreover, treatment with bicalutamide reverses the AR binding and abrogates the effects of AR on cell proliferation. Taken together the data suggests that AR mediated chemopreventive effect can be reversed by AR antagonist like bicalutamide and therefore we propose to use bicalutamide for the treatment of AR positive, triple negative breast cancer.

## Discussion

Specific binding of transcription factors to DNA is one of the most common ways by which gene expression is controlled. Computational evaluation of protein-DNA interaction is important for the identification of DNA-binding sites. Moreover, binding affinity calculations can be very instrumental in the validation of the predicted binding residues between protein and the DNA. Docking was carried out to obtain the most likely orientation of B DNA on the AR-DNA binding site. The maximum conformers with lowest energy pose of gene were chosen for further analysis by performing interaction mapping between AR-DBD and ARE(s). Our data shows that AR-DBD has two distinct DNA binding sites, indicating that the behavior of AR protein is not specific, albeit the lowest energy pose of AREs tends to orient themselves quite similarly (Fig. 4A and 4B). The electrostatic surface representations were found to be in agreement with the docking results as best fit pose of DNA showed interaction with the basic residue of AR-DBD localized at the surface (Fig. 4C). Our data suggest that guanine and cytosine were the most conserved residues in alignment of all the AREs (Fig. 5A). We further propose that in a typical ARE, the position of the conserved guanine or cytosine can be at position 2/3 and 10/11. In this study with the help of computational docking tool, we observed that K567, K588, R591 and K592 are the novel residues found to be involved in the DBD and ARE(s) interaction (Table 2, Fig. 5B-F). Recently Helsen C. et al have shown that mutation of residue K592 significantly reduced the transactivation function of AR. We speculate that the residues K567, K588, R591 and K592 are not only involved in the stabilization of the binding between AR-DBD and ARE but they also regulate the transactivation function of AR. It remains plausible that additional residues involved in the DNA-protein interaction might provided a graded affinity to response element as well as the transactivation function. However, It is a limitation of the study that docking should be ideally validated by making AR DBD mutants and to observe the docking of the mutant AR to the cognate ARE sequences.

**Fig 9:**
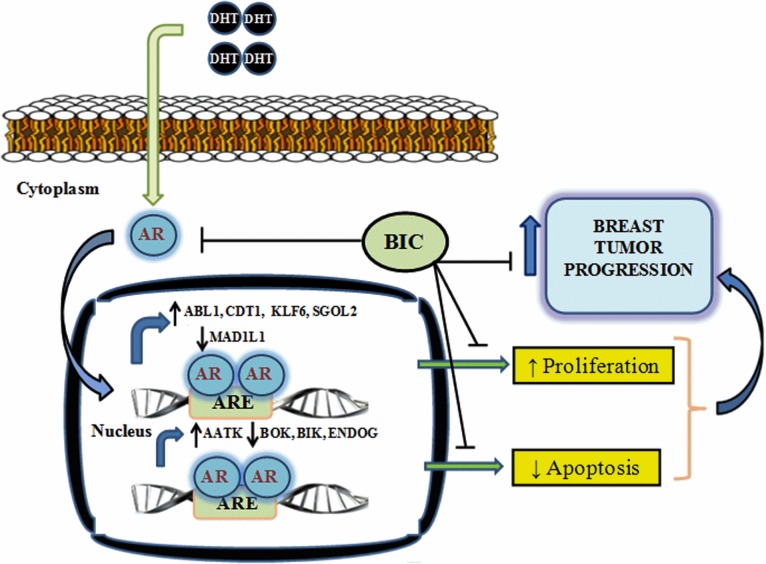
Proposed model showing AR regulation of the breast tumour progression. Androgen receptor (AR) on DHT stimulation translocates into the nucleus and bind to its cognate androgen response elements (AREs). Inside the nucleus, AR up regulates the expression of ABL1, CDT1, KLF6 and SGOL2, while it represses MAD1L1 expression to induce cell proliferation. At the same it induces AATK expression and down regulates the expression of BOK, BIK and ENDOG to decrease apoptosis and promotes breast cancer progression. Bicalutamide (BIC) reverses the effect of AR on cell cycle and apoptosis by binding and preventing its activation. Due to its ability to negate the effects of AR, bicalutamide can be used to block breast cancer progression.

Recent literature suggests that AR is emerging as a interesting target for breast cancer therapy in ER-/AR+ tumors and some triple negative breast cancers (TNBCs), which are enriched for AR signalling pathways [12,61]. Due to the presence of a few targeted therapies for the TNBCs, there is an urgent need for studying the molecular mechanism involved in such types of breast cancers. It was observed that in the presence of chemotherapeutic drugs like paclitaxel, 5-fluorouracil and cyclophosphamide, addition of DHT reverses the chemotherapeutic drug-induced apoptosis (Fig. 7A). However, this chemo preventive effect of AR (on DHT addition) was reversed by addition of bicalutamide (Fig. 7A). Similar resistance to chemotherapeutic drug paclitaxel had been observed in ER+ BCap37 cells, where it was proposed that expression of ERα mainly interferes with paclitaxel-induced apoptotic cell death [62]. In the present study, it was found that AR induces cell proliferation in MDA-MB-453 cell in the presence of DHT and this effect was countered by the addition of bicalutamide (Fig. 7E-F). AR is known to promote cell proliferation in prostate cancer in androgen dependent manner [63]. Moreover, it was observed that AR mediates a chemopreventive effect on the drug induced apoptosis and it suppresses drug induced apoptosis in a DHT dependent manner (Fig. 8A,B). Therefore AR antagonists can be used for the therapeutic intervention of drug resistant AR positive, triple negative cancer. AR mediates cell proliferation by directly binding to the promoters of CDT and KLF6 on DHT stimulation (Fig. 8C-D and S2 Fig.). AR was completely stopped on addition of bicalutamide or the IC_50_ concentration of the drug. Bicalutamide is known to prevent nuclear localization of AR and therefore no binding was observed in the presence of bicalutamide in combination with a drug or bicalutamide alone (Fig. 8C-D and S2 Fig.). However, we observed binding in presence of the drug, DHT and bicalutamide suggesting that binding was due to some residual AR present in the nucleus. The consensus sequence of the hormone response element (HRE) of the three other prominent nuclear receptors namely glucocorticoid receptor (GR), progesterone receptor (PR), and AR are very similar to each other. The binding to the target ARE's is not only shared between the nuclear steroid receptors but the binding can also deviate considerably from the consensus sequence [63,64]. It is postulated that specificity of the recognition of the cognate HRE is done by both the sequences flanking the HRE and the associated complexes of the accessory proteins. Glucocorticoid receptor (GR) is a steroid nuclear receptor which is known to up regulate a set of androgen responsive genes in AR-independent manner and it can by bypass the AR in regulation of some of the AR targets[65]. Therefore AR targeting in the clinical settings may be affected by this process and combined targeting of AR and GR might be the ideal therapy for the AR positive, triple negative breast cancer. Taken together our data shows that AR upon stimulation by androgens such as DHT leads to induction of cell cycle and it represses chemotherapeutic drug-induced apoptosis. We postulate that stimulation with DHT, AR translocates to the nucleus and induces the cell cycle genes like ABL1, KLF6, SGOL2 and CDT1 whereas it represses MAD1L1. ABL1 is a protoncogene involved in a variety of cellular processes, including cell division, adhesion, differentiation, and response to stress. CDT1 is overexpressed in many tumour derived cell lines and its overproduction can lead to carcinogenesis by inducing re-replication and chromosomal damage in normal cells [66]. SGOL2 promotes chromosomal segregation during the cell division. AR up regulates ABL1 along with SGOL2, KLF6 and CDT1 to promote cell proliferation. Kruppel like factors (KLFs) are DNA-binding transcriptional regulators and their expression is altered in human cancers. Tumor derived KLF6 mutants and alternatively spliced isoforms promote tumorigenesis and they potentiate oncogenic WNT signalling[67]. AR represses MAD1L1, which functions as tumor suppressor to positively influence proliferation. It was observed that AR downregulates all the apoptotic genes except AATK, to decrease apoptosis. Our data shows that AATK is up regulated by DHT treatment, Recent study showed that overexpression of AATK inhibits cell proliferation and promotes apoptosis but over expression of wildtype AATK was found to promote neurite out growth[68]. Literature suggested that AR signalling promotes outgrowth of neurite and also the neurite like outgrowth of breast cancer cells promotes tumour growth and metastasis [69,70]. These data indicate that AATK may have cancer promoting activity in a tissue specific manner. Moreover the effects of DHT on cell proliferation and apoptosis are reversed by the treatment of breast cancer cells with bicalutamide and we propose that it might be useful in the chemotherapy of TNBCs, which are positive for downstream AR signalling (Fig. 9). Thus we propose the model that AR on DHT stimulation promotes proliferation and decreases cell death to help in the process of breast cancer progression (Fig. 9). Our data is further supported by the findings that recently there is a clinical trial (NCT00468715) underway testing the effect of bicalutamide in preselected patients with ER-/ AR+ tumors [13,61]. Interestingly, bicalutamide addition to MDA-MB-453 breast cancer cells caused a highly significant increase in the paclitaxel-induced cell death (Fig. 7A). The action of paclitaxel and bicalutamide is partly supported by the findings that taxanes are known to inhibit the androgen-independent activation of AR by the action of FOXO1 in prostate cancer [71]. In TNBCs, paclitaxel along with anthracyclines are the chemotherapeutics of choice. Moreover, due to resistance to paclitaxel and anthracyclines, there is an urgent need for novel targeted therapies for the treatment of TNBCs[72]. Furthermore, we propose that taxane therapy along with bicalutamide can be used effectively for the treatment of TNBCs, which are positive for downstream AR signalling pathway. In summary, our work provides better understanding of the process of AR recognition of its cognate AREs. Collectively the data enhances our understanding of AR function in TNBCs and it provides novel targets for the therapeutic intervention of triple negative breast cancer, which are positive for downstream AR signalling pathway.

## Supporting Information

S1 Fig Androgen regulation of the target genes in LNCaP (A) ChIP assay were performed with AR antibody or control IgG antibody in LNCaP cells treated with 10nM/L of DHT or vehicle control for 24 hrs. The fold enrichment of coprecipitating DNA was determined by qPCR for the indicated promoters. Error bars are means ± SD of three independent experiments; (*p < 0.05, **p < 0.01, ***p < 0.001). (B) Standard PCR for the AR, IgG and input DNA was performed for the indicated genes.
(TIF)

S2 Fig AR binding to the promoter of CDT and KLF (A-B) ChIP-qPCR showing the binding of AR to CDT promoter in presence of cyclophosphamide and 5'-Fluorouracil (C-D) ChIP-qPCR showing the binding of AR to KLF promoter in presence of cyclophosphamide and 5'-Fluorouracil. All the means and standard deviation are obtained from three independent experiments. Gel picture below shows the standard PCR validation of the result Cyclo: Cyclophosphamide; Fluoro: 5'-Fluorouracil, M: Marker, 1: Input, 2: Untreated control, 3: DHT +Pac,: 4: DHT+ Bic +Pac, 5: Bic + Pac, 6: Bic.
(TIF)

S1 Table List of primer pairs used for Real time quantitative PCR. (DOC)

S2 Table (A). List of primer pairs used for quantitative ChIP qPCR. (B). List of internal primer pairs used for quantitative ChIP qPCR. (DOC)
