# Supplementary figures and images for: A Molecular Analysis Provides Novel Insights into Androgen Receptor Signalling in Breast Cancer
Source: PLoS One. 2015 Mar 17;10(3):e0120622. doi: 10.1371/journal.pone.0120622 (PMC4364071; doi:10.1371/journal.pone.0120622)

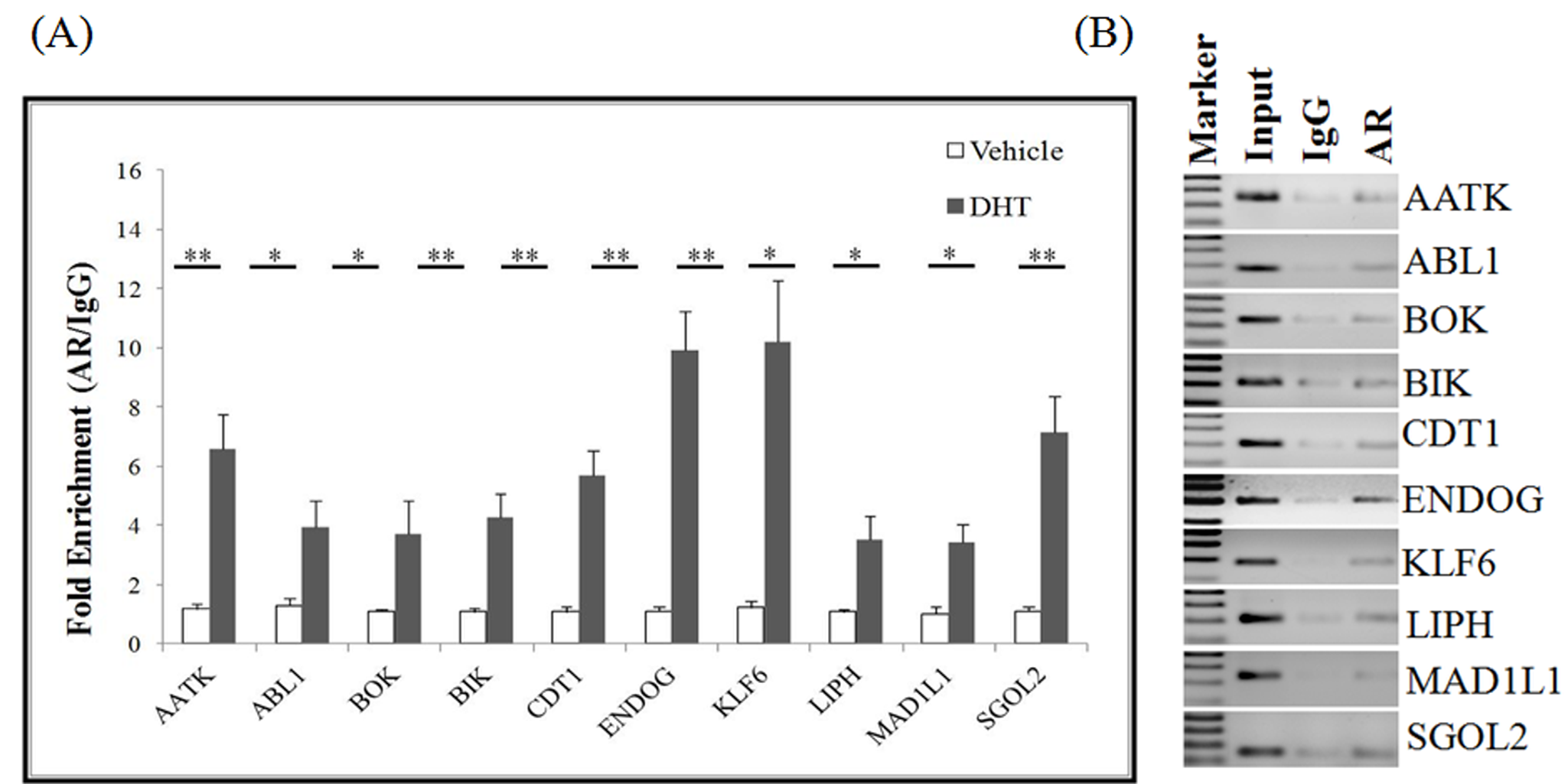

Supplement: S1 Fig — (A) ChIP assay were performed with AR antibody or control IgG antibody in LNCaP cells treated with 10nM/L of DHT or vehicle control for 24 hrs. The fold enrichment of coprecipitating DNA was determined by qPCR for the indicated promoters. Error bars are means ± SD of three independent experiments; (*p < 0.05, **p < 0.01, ***p < 0.001). (B) Standard PCR for the AR, IgG and input DNA was performed for the indicated genes. (TIF) [file pone.0120622.s001.tif]

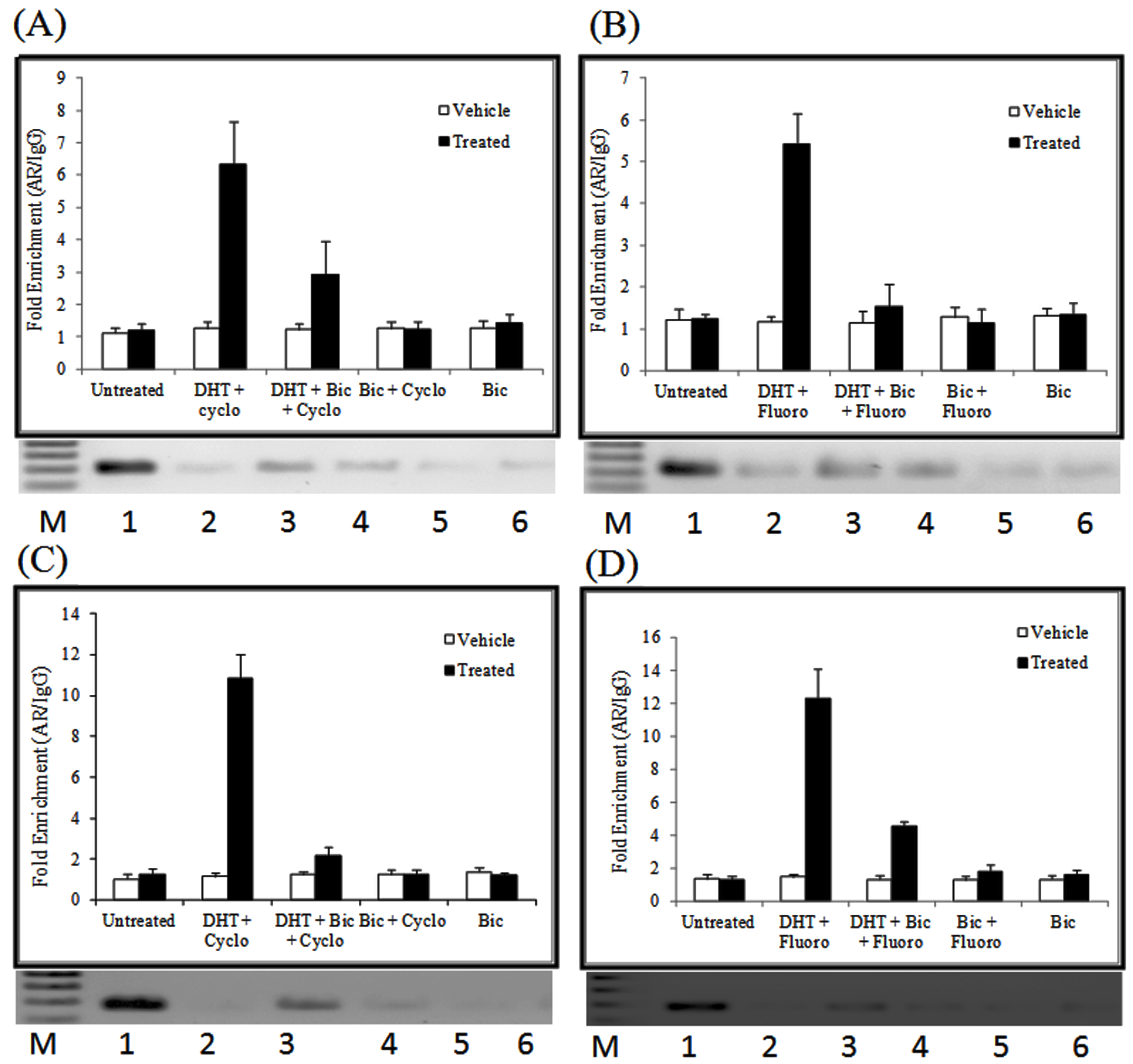

Supplement: S2 Fig — (A-B) ChIP-qPCR showing the binding of AR to CDT promoter in presence of cyclophosphamide and 5'-Fluorouracil (C-D) ChIP-qPCR showing the binding of AR to KLF promoter in presence of cyclophosphamide and 5'-Fluorouracil. All the means and standard deviation are obtained from three independent experiments. Gel picture below shows the standard PCR validation of the result Cyclo: Cyclophosphamide; Fluoro: 5'-Fluorouracil, M: Marker, 1: Input, 2: Untreated control, 3: DHT +Pac,: 4: DHT+ Bic +Pac, 5: Bic + Pac, 6: Bic. (TIF) [file pone.0120622.s002.tif]
