# Supplementary material for: A Molecular Analysis Provides Novel Insights into Androgen Receptor Signalling in Breast Cancer
Source: PLoS One. 2015 Mar 17;10(3):e0120622. doi: 10.1371/journal.pone.0120622 (PMC4364071; doi:10.1371/journal.pone.0120622)
Supplement: S1 Table — (DOC) [file pone.0120622.s003.doc]

**Table S1. List of primer pairs used for quantitative PCR**

| Gene Name | Gene ID | Abbreviation | Primer sequence |
| --- | --- | --- | --- |
| Apoptosis-associated tyrosine kinase | 9625 | AATK | FP: 5'-AGCAGTGAGGATGAGGACAC-3'  RP: 5'-GCCTCCTTGAGCACAAACTC-3' |
| c-abl oncogene 1, non-receptor tyrosine kinase | 25 | ABL1 | FP: 5'-AAGCCGCTCGTTGGAACTC-3'  RP: 5'-AGACCCGGAGCTTTTCACCT-3' |
| BCL2-interacting killer | 638 | BIK | FP: 5'-GACCTGGACCCTATGGAGGAC-3'  RP: 5'-CCTCAGTCTGGTCGTAGATGA-3' |
| BOK BCL2-related ovarian killer | 666 | BOK | FP: 5′-GGCGATGAGCTGGAGATGAT-3′  RP: 5′-ACACTTGAGGACATCAGTCC-3' |
| chromatin licensing and DNA replication factor 1 | 81620 | CDT1 | FP: 5'-CGGTGGACGAGGTTTCCAG-3'  RP: 5'-CTGCCGGGGTGGATTTCTT-3' |
| Endonuclease G | 2021 | ENDOG | FP: 5′-CGACACGTTCTACCTGAGCA-3′  RP: 5′-AGGATTTCCCATCAGCCTCT-3′ |
| KLF6 Kruppel-like factor 6 | 1316 | KLF6 | FP: 5'-GGCAACAGACCTGCCTAGAG-3' RP: 5'-CTCCCGAGCCAGAATGATTTT3' |
| Lipase, member H | 200879 | LIPH | FP: 5'-CACGATCTCTCCAGTTTCCA-3' RP: 5'-AAAGCTCAGCCTGGTGAATG-3' |
| Mitotic arrest deficient-like 1 | 8379 | MAD1L1 | FP: 5'-GAGATGAGAGAGACCAACGGG-3' RP: 5'-GCTCAACCACGAATCTGGAAA-3' |
| Shugoshin-like 2 | 51246 | SGOL2 | FP: 5'-TAAAGCACAACAACAGGGCAT-3'  RP: 5'-'AGGCGAAGAAATGTGTTCTCAAA-3' |
