# Supplementary material for: A Molecular Analysis Provides Novel Insights into Androgen Receptor Signalling in Breast Cancer
Source: PLoS One. 2015 Mar 17;10(3):e0120622. doi: 10.1371/journal.pone.0120622 (PMC4364071; doi:10.1371/journal.pone.0120622)
Supplement: S2 Table — (DOC) [file pone.0120622.s004.doc]

**Table S2 (A). List of primer pairs used for quantitative ChIP qPCR**

| **Gene Name** | **Gene ID** | **Primer name** | **Primer Sequence** |
| --- | --- | --- | --- |
| AATK | 9625 | AATK FP  AATK RP | 5'-TATGTCACCCCAAACCCATGC -3'  5'-GGATCCTTCCTGCCTCTCCG-3' |
| ABL1 | 25 | ABL FP  ABL RP | 5'-CCGGCAAGAATCTACCAGGTC-3'  5'-GGAGCAGTAGGGTTGGATCTC-3' |
| BIK | 638 | BIK FP  BIK RP | 5’-AGTGCAGTGGTGTGATCTCA-3'  5'-CGGATCACAAGGTCAGGAGT-3' |
| **BOK** | 666 | BOK FP  BOK RP | 5'-CGGTGCCATCTCACTGCT-3'  5'-CCCCTGCAGGAAACCAGG-3' |
| CDT1 | 81620 | CDT FP  CDT RP | 5'-AGACGGAGTCTCGCTGTC-3'  5'-CCATCCTGGCTAACACGGTG-3' |
| ENDOG | 2021 | ENDOG FP  ENDOG RP | 5'-CTCTGGGTGGTGGAGCAG-3'  5'-GTGTCGTCCATGGCCTTCTG-3' |
| **KLF6** | 1316 | KLF FP  KLF RP | 5'-GGCGAAGTTTTGGTCAGCTC-3'  5'-TCGCCATTTCCCTTGTCACC-3' |
| LIPH | 200879 | LIP FP  LIP RP | 5'-ATGGAGTCTTGCTCTGTC-3'  5'-GTGGTGAAACCTCATCTCT-3' |
| MAD1L1 | 8379 | MAD FP  MAD RP | 5'-AACGGAGTCTTGCTCTGG-3'  5'-CTGTAATCCCAGCACTTTGTG-3' |
| SGOL2 | 151246 | SGOL FP  SGOL RP | 5'-CTGGGACTGCTCTACTCCCT-3'  5'-GTCTTCCATCACCCCTTCGC-3' |

**Table S2 (B). List of internal primer pairs used for quantitative ChIP qPCR**

| **Gene Name** | **Gene ID** | **Primer Name** | **Primer sequence** |
| --- | --- | --- | --- |
| AATK | 9625 | AATK FP | 5’AGGCAAGCAGACCCTACAGA3’ |
|  |  | AATK RP | 5’ATGTCACAAGCACACCCAAA3’ |
| ABL1 | 25 | ABL FP | 5’CCATAGGAATGTGTCGGGCA3’ |
|  |  | ABL RP | 5’ACCAAAAAGGCAGGGACGAA3’ |
| BIK | 638 | BIK FP | 5’AGATCCCTGACCCTGACTTG3’ |
|  |  | BIK RP | 5’AACCAGGCATCTCAAACCCAA3’ |
| **BOK** | 666 | BOK FP | 5'GTGTCCCTGTATGCGGTGG3' |
|  |  | BOK RP | 5'CACACGGGCACTCAGGC3' |
| CDT1 | 81620 | CDT FP | 5'GAGCGTCTTTGTGTCCGAACG3' |
|  |  | CDT RP | 5'AGAACAGATCAGTGACAGACACC3' |
| ENDOG | 2021 | ENDOG FP | 5'CCTCTGTGGGGAGAGATGGA 3' |
|  |  | ENDOG RP | 5'TCCTCCTTGAGGCTGGAACT3' |
| **KLF6** | 1316 | KLF6 FP | 5'CCTGTCCCTGAAGACACTGATG3' |
|  |  | KLF6 RP | 5'GGGCAAATCAGAAGCACAGAAG3 |
| LIPH | 200879 | LIPH FP | 5'TCCAGTGAACCCACCACATT3' |
|  |  | LIPH RP | 5'AAACCGTACCCTCAGCTCAC3' |
| MAD1L1 | 8379 | MAD1L1 FP | 5'ATCGTTTCCATTTCCTGCGTG3' |
|  |  | MAD1L1 RP | 5'CTGCTGAGGGTCCAAGAGAA3' |
| SGOL2 | 151246 | SGOL 2FP | 5'GACCTTCCTGATAACTCCTACCAC3' |
|  |  | SGOL2 RP | 5'GTGATGACCATCCAGTTGCCA3' |
